# Oh So Sweet: A Comparative Investigation of Retail Market Composition of Sweetened and Flavoured Beverages in Singapore and Australia

**DOI:** 10.3390/nu15010247

**Published:** 2023-01-03

**Authors:** Kim Anastasiou, Paige G. Brooker, Xenia Cleanthous, Rebecca Tan, Benjamin P. C. Smith, Malcolm Riley

**Affiliations:** 1 Human Health, CSIRO Health and Biosecurity, SAHMRI, North Terrace, Adelaide 5000, Australia; 2 Singapore Institute of Food and Biotechnology Innovation, Agency for Science, Technology and Research, Singapore 138669, Singapore; 3 Future Ready Food Safety Hub, C/O School of Chemistry, Chemical Engineering and Biotechnology, Nanyang Technological University, Singapore 637459, Singapore

**Keywords:** beverage composition, sugar-sweetened beverages, non-nutritive sweeteners, Health Star Rating, Healthier Choice Symbol, front-of-pack label, Nutri-Grade, Singaporean food supply, Australian food supply

## Abstract

The consumption of sugar and non-nutritive sweeteners has been associated with poor health outcomes. The aim of this paper was to provide a comparison of the range of sweetened or flavoured beverages between two high-income countries in the Asia-Pacific region: Australia and Singapore. Following the FoodTrack^TM^ methodology, nutrition, labelling, and price data were collected from major Australian and Singaporean supermarket chains and convenience stores. The nutrient profiles of products were tested for differences using Kruskal–Wallis and Mann–Whitney U tests. The greatest number of products collected in Australia were from the ‘carbonated beverages’ category (*n* = 215, 40%), and in Singapore the greatest number of products were from the ‘tea and coffee ready-to-drink’ category (*n* = 182, 35%). There were more calorically sweetened beverages in Singapore compared with Australia (*n* = 462/517 vs. n = 374/531, *p* < 0.001). For calorically sweetened products, the median energy of Singaporean products was significantly higher than Australian products (134 kJ vs. 120 kJ per 100 mL, *p* = 0.009). In Australia, 52% of sweetened or flavoured beverages displayed a front-of-pack nutrient signposting logo, compared with 34% of sweetened or flavoured beverages in Singapore. These findings also indicate that the consumption of just one serving of calorically sweetened carbonated beverages or energy drinks would exceed the WHO maximum daily free sugar recommendations.

## 1. Introduction

Individuals’ food choices are driven by a range of factors, including affordability, accessibility [1], palatability, cultural traditions, cognitive factors (stress, anxiety, and attitudes towards health) and physiological feedback mechanisms [2]. Humans’ inherent desire for sweetness has helped food choice to meet energy requirements throughout history [3]. Frequent consumption of sweet foods and beverages can reinforce and shape taste preferences starting from infancy [3,4].

For the food industry, sugar is a cheap flavour enhancer and also functions as a preservative, texture modifier, fermentation substrate, colouring agent, and bulking agent [5]. However, high consumption of added sugar is associated with poor health outcomes such as cardiovascular disease [6], type 2 diabetes [7] and dental caries [8]. Guidelines from the World Health Organization (WHO) recommend adults and children limit their intake of ‘free sugars’ (defined as free sugars added to foods and beverages or naturally present in honey, syrups, fruit juices, and concentrates) to <10% of total energy, with an intake <5% providing additional benefits [8]. To achieve this, they suggest “limiting the consumption of foods and drinks containing high amounts of sugars, such as… sugar-sweetened beverages…” [9]. 

Sugar-sweetened beverages (SSBs) and drinks that use non-nutritive sweeteners (NNS) are an example of (often ultra-processed) food products that provide consumers with sweetness and stimulate receptors in the brain to elicit a pleasure response [10]. SSBs are the largest source of added sugar in diets across the globe and a major contributor to dietary energy [11]. In the western Pacific region, the mean consumption of SSBs by children (aged 2–18 years) over the last decade was estimated to be 298 mL/day [12]. The overconsumption of SSBs is driven by their prolific availability [13], low cost [14] and variety of products available [15]. 

Unlike other sugar-containing foods, sweetened beverages are inherently discretionary; that is, they do not add any necessary nutritional value to a diet beyond hydration. Thus, reducing SSB consumption has become the target of many public health policy interventions, including front-of-pack labels and sugar taxes. Many manufacturers have responded to this by offering a wide range of beverages sweetened with NNS. Artificially sweetened products using NNS have generally been accepted as a safe and efficacious way to reduce overall caloric consumption and avoid blood glucose elevation [16], particularly from foods which are not intended to be core components of dietary intake [17,18]. However, concerns have been raised about their impacts on the gut microbiome [19] and diseases such as cancer [20]. Taken together, the balance of the total evidence on the health effects of NNS is weak and inconclusive [21].

Additionally, concerns have been raised that ongoing sweetening of the food supply [22], aided by frequent consumption of NNS beverages may encourage a predisposition to enjoy other calorically sweetened foods [4,23,24]. However, recent evidence is not supportive of this concern [25,26,27]. Concerns have also been raised about the potential impacts on human and ecosystem water supplies because some NNS are not removed by standard wastewater treatment [28,29]. Recently proposed WHO guidelines suggest “that NSS not be used as a means of achieving weight control or reducing risk of noncommunicable diseases” [30]. However, this is a conditional recommendation, graded with low overall certainty when assessed according to the GRADE criteria [30].

Analysing the differences and similarities between national food supplies is useful to facilitate consistent and effective food standards and harmonise regulations for a healthy global food supply. Australia and Singapore are two high-income countries in the western Pacific region. According to the most recent Australian National Dietary Intake Survey (2011–2012), SSBs account for 35% of total added sugars in adults’ diets [31]. In Singapore, the Health Promotion Board’s 2018 survey showed that, on average, Singaporeans consumed twelve teaspoons (or 60 g) of sugar daily, with over half being attributed to sugar-sweetened beverages (SSBs), of which 64% were pre-packaged SSBs [32]. A recent study by Tan et al. (2021) showed that over half (59%) of non-alcoholic beverages on the Singapore market contained sugar and were considered ‘less healthy’, defined as Nutri-Grades of ‘C and D’ [33]. The Nutri-Grade system is a new product labelling requirement to aid consumers to manage their sugar and fat consumption and improve the overall nutritional quality of the food supply [34,35]. Accurate, consolidated, nationally representative datasets of the retail food supply are valuable tools that can be used to justify the need for and monitor the effectiveness of policies such as front-of-pack labelling, reformulation, and even fiscal policies. 

Another influence on individuals’ food choices is the promotion of products, such as their front-of pack-labelling [36]. Front-of-pack nutrition labels (FOPLs) provide consumers with a quick and easy-to-interpret way to compare the nutritional compositions of similar packaged foods. Many governments have supported the implementation of voluntary FOPLs. However, these are not standardised across countries. 

In 2014, the Australian government introduced the Health Star Rating system (HSR), a voluntary FOPL system that rates the overall nutrient profiles of packaged foods and assigns a rating from 0.5 to 5 stars [37]. The system is intended as a simple way for consumers to interpret the nutrient composition of foods, and thus guide consumers to make healthier choices [37]. Similarly, in 2001 the Singaporean Government introduced a voluntary FOPL system: the Healthier Choice Symbol (HCS). The HCS is displayed on food products that meet select nutrient criteria [38].

The aim of this paper was to provide a comparison of the market availability of sweetened or flavoured beverages between two countries: Australia and Singapore. The compared data included product variety and availability, nutrient composition, and the uptake of voluntary labelling systems. The data analysed from each country can be used to inform future policies and can act as a baseline measurement for upcoming policy changes. In addition, the comparison of two food supplies and their FOPL policies may help to clarify and align international food policies in an increasingly globalised food system.

## 2. Materials and Methods

### 2.1. Data Collection Methods

This analysis describes the availability and characteristics of sweetened or flavoured beverages in Australia and Singapore using data collected using the FoodTrack^TM^ methodology. In collaboration with the National Heart Foundation of Australia, the FoodTrack^TM^ database was developed by the Commonwealth Scientific and Industrial Research Organisation (CSIRO) in 2014. Designed to monitor the Australian supermarket food supply, FoodTrack^TM^ consists of a custom-designed data collection app, a remote database, and a web portal to record nutrition, labelling, and cost information of packaged food and beverages sold in supermarkets. In Australia, data were collected from the four major supermarket chains: two major metropolitan locations of Coles, Woolworths, Aldi, and IGA (eight stores in total). Details on data collection were previously described in more detail [39]. In Singapore, data were collected for a pilot study from the four major retail supermarkets, Cold Storage, Giant, NTUC FairPrice, and Sheng Siong, and the two major convenience stores, 7-Eleven and Cheers (12 stores in total). Based on an assessment of the market share and product listings in both countries, it was estimated that these stores covered >80% of the beverage types sold in the Australian and Singaporean markets [40]. Data collection for FoodTrack™ was suspended for 2020 in Australia due to the COVID-19 pandemic, but data were collected in Singapore. Therefore, 2019 Australian data were compared with 2020 Singaporean data.

### 2.2. Data Cleaning and Preliminary Analysis

Data were cleaned and analysed by trained nutrition researchers. Duplicate products (products sold in multiple pack sizes or which contained more than one NIP in the FoodTrack^TM^ database) were identified. One pack size was retained per flavour variant. For all products, including concentrated products (such as sports powders and cordial syrups), NIP data for the products as prepared according to manufacturer instructions were used. If these data were not available, products were excluded from the analysis. The maximum and minimum nutritional values were checked and compared to the pack images to ensure accuracy. 

NIP data for products where data were displayed ‘per serving’ were standardised to per 100 mL or 100 g, depending on the form of the food. All energy values displayed on-pack in kilocalories were converted to kilojoules using a factor of 4.2. Values listed on the NIP as ‘less than’ were adjusted to their closest whole number for analysis. For example, sodium < 5.0 mg/100 g was adjusted to 5.0 mg/100 g.

Products were classified into six primary categories and further into two subcategories within each of these (Table 1). In this study, sweetened or flavoured beverages excluded plain water, fruit juice, and flavoured milks.

The data included at each stage of the project are described in Supplementary Figures S1 and S2.

### 2.3. Statistical Analysis

Following data cleaning in Microsoft Excel 365, data were analysed using the Statistical Package for Social Sciences (SPSS) version 26 (IBM, New York, NY, USA). Cross-country comparisons between Australia and Singapore were conducted by category and subcategory. 

For each country, the number (n) and proportion (%) of products were calculated for each category and subcategory. Data were tested for normality using the Shapiro–Wilk test, accompanied by visual inspections of Q-Q plots. Data were not normally distributed, and therefore non-parametric tests were applied. Unless otherwise stated, data are presented as medians ± inter-quartile ranges (IQR).

Descriptive statistics were calculated to describe the proportions of products displaying selected front-of-pack labels (FOPLs; HSR, HSR kJ symbol, Nutri-Grade, and HCS) and the medians and IQRs of the HSR scores. FOPL percentages are displayed according to the number of eligible products, noting that all Australian products were eligible for the HSR, according to the 2019 HSR guidelines [41], but energy drinks were not eligible for the Singaporean HCS [42]. Descriptive statistics describing the medians ± IQRs for energy (kJ/100 g or kJ/100 mL) and total sugar content (g/100 g or g/100 mL) were generated for each subcategory in Australia and Singapore. Differences in the proportions of products within categories across countries were examined by chi-square tests. To determine differences in the median energy and total sugar content across countries and the median HSR rating between calorically and non-calorically sweetened beverages, Mann–Whitney U tests were conducted. Kruskal–Wallis tests were used to determine the differences in the median energy and sugar content of beverages between categories within each country. 

In addition to the above analyses, the findings were compared with the World Health Organization (WHO) guidelines on free sugars. The WHO guidelines outline two recommendations: that free sugars should contribute < 10% of total dietary energy and that additional health benefits are found when free sugars contribute < 5% of total dietary energy. To determine the potential contribution of free sugars within the products to dietary energy intakes, the medians and IQRs for each product category were calculated according to the following:

Percent contribution of one serving of the beverage to the WHO guidelines;
Millilitres of drink that would need to be consumed to exceed the WHO guidelines;
Servings of drink that would need to be consumed to exceed the WHO guidelines.

Statistical analyses were conducted with SPSS version 26. Statistical significance was set at *p* < 0·05.

## 3. Results

Of the 1604 relevant products identified in the FoodTrack^TM^ database for Australia and Singapore, 1054 met the inclusion criteria. Supplementary Table S1 shows a detailed breakdown of the excluded products. Of note is that 30 Singaporean vinegar drinks did not display the nutrient composition of the ‘as consumed’ products and therefore were excluded from further analyses. 

### 3.1. Numbers and Types of Products Available in Singapore and Australia

Similar numbers of flavoured or sweetened beverages were recorded in Australia and Singapore (*n* = 531 vs. 523, respectively, see Table 2). In Australia, the greatest number of collected products were from the ‘carbonated beverages’ category (*n* = 215, 40%), followed by ‘flavoured waters and cordials’ (*n* = 132, 25%) and ‘tea and coffee RTD’ (*n* = 93, 18%). In comparison, the greatest number of products collected in Singapore were from the ‘tea and coffee RTD’ category (*n* = 182, 35%), followed by ‘flavoured waters and cordials’ (*n* = 158, 30%) and ‘carbonated beverages’ (*n* = 131, 25%). ‘Drinking vinegars’ and ‘alcohol alternatives’ were the least prevalent in both Australia (*n* = 4, 1%; *n* = 11, 2%, respectively) and Singapore (*n* = 8, 2%; *n* = 1, <1%, respectively). 

The relative distributions of products across the seven categories were different between Australia and Singapore. Proportionally, there were more carbonated beverages (χ(1) = 33.898, *p* < 0.001) and sports drinks (χ(1) = 8.210, *p* = 0.006) in Australia (40% and 9%, respectively) compared to Singapore (25% and 5%, respectively, see Table 2). There was a greater proportion of ‘tea and coffee RTD’ products in Singapore (18%) than Australia (35% (χ(1) = 26.902, *p* < 0.001)). The proportions of energy drinks and flavoured waters and cordials were similar between Australia and Singapore. However, when further broken down into calorically versus not calorically sweetened, there were significantly more calorically sweetened flavoured waters and cordials in Singapore (99%) versus Australia (79%) (χ(1) = 30.850, *p* = <0.001). 

Across both countries, there were consistently more calorically sweetened products within each category, compared with not calorically sweetened products, with the exception of ‘drinking vinegars’. 

### 3.2. Energy and Sugar Content

In Australia, energy and total sugar values were displayed on-pack for all 531 products. Of the 523 products in Singapore, 37 (7%) did not display an energy value and 54 (10%) did not display a total sugar value. 

The results for the calorically sweetened beverages (*n* = 836, 79% of total products) are presented in Table 3. The non-calorically sweetened beverages, by definition, contained <1 g of total sugar and contained little energy; the nutritional compositions of non-calorically sweetened products are provided in Supplementary Table S2. 

For calorically sweetened products, the median energy of Singaporean products was significantly higher than that of Australian products (134 ± 80 vs. 120 ± 98 kJ per 100 mL, *p* = 0.009). While sugar was also higher in Singapore compared with Australia, this difference was not significant (6.8 ± 4.2 vs. 6.4 ± 6.1 g sugar per 100 mL, respectively, *p* = 0.129). When analysed according to product category, the median energy and sugar content of beverages in Australia versus Singapore were similar for most categories (see Table 3). Statistically significant differences were observed for three categories: Singaporean ‘flavoured waters and cordials’ and ‘tea and coffee RTD’ contained more energy and sugar than similar products found in Australia; while Australian sports drinks contained significantly more energy than Singaporean sports drinks (see Table 3). 

### 3.3. Free Sugars Present in Sweetened or Flavoured Beverages Compared to WHO Consumption Guidelines

Current World Health Organization (WHO) guidelines recommend the consumption of no more than 10% of total daily energy from free sugars to mitigate health risks associated with their consumption [8]. The median contribution for one serving (as recommended by the manufacturers) of calorically sweetened beverages in both Australia and Singapore is 34% of the WHO recommendation benchmark (based on a daily intake of 8700 kJ; Table 4). The WHO notes added health benefits if free sugars contribute <5% of total daily energy. According to this cut-off, the median contributions for one serving of products in Australia and Singapore would be 69% and 68% of the recommended maximum daily total free sugar intake, respectively. In fact, the median energy per one serving of a calorically sweetened carbonated beverage (Singapore and Australia) or an energy drink (Australia) was enough to exceed this second WHO guideline (see Table 4). 

The median number of millilitres of each calorically sweetened product category required to be consumed to exceed the WHO guidelines was also calculated. Of note is that between 233 and 298 mL of carbonated beverages and energy drinks would meet the recommended maximum intake of free sugars according to the stricter WHO guidelines (<5% of total daily energy), which is comparable to some of the can sizes in the FoodTrackTM database. 

### 3.4. Front of Pack Labelling

**Figure 1:**
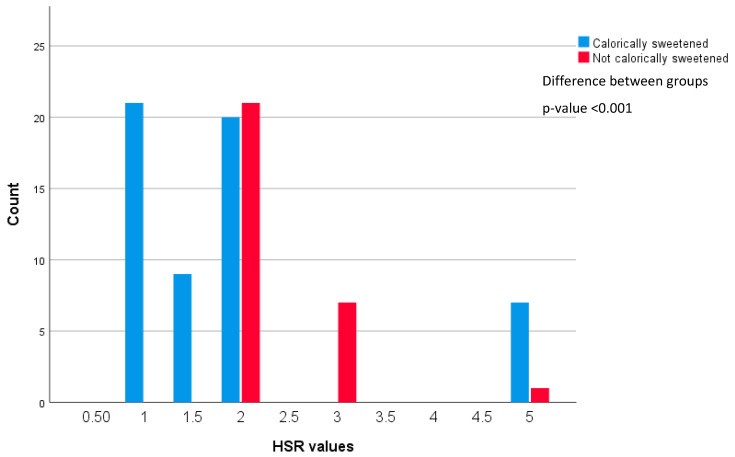
Distribution of HSR values displayed on sweetened or flavoured beverages in Australia.

In Australia, half of the products included in this analysis (*n* = 274, 52%) displayed either the HSR score or HSR kJ icon. Approximately one third (*n* = 86, 16% of total products) displayed an HSR score (0.5 to 5, in 0.5 increments), and the remainder displayed the energy (kJ) icon, see Table 5. Of those displaying an HSR score, the majority displayed HSR scores below 2.5 (see Figure 1). HSR scores were most frequently found on ‘carbonated beverages’ and ‘flavoured waters and cordials’ (*n* = 38, 18%; *n* = 36, 27%, respectively). ‘Flavoured waters and cordials’ were the only products to display an HSR ≥ 3.5 (*n* = 8 products; *n* = 7 calorically sweetened, n = 1 non-calorically sweetened), which has previously been used to differentiate products that score poorly from those with more acceptable nutrient contents [43]. There were few differences between the calorically sweetened and non-calorically sweetened products. However, the median values for non-calorically sweetened ‘carbonated beverages’ and ‘flavoured waters and cordials’ displaying the HSR were slightly higher than their calorically sweetened counterparts (see Table 5).

Compared to the products in Australia displaying the HSR symbol (excluding the kJ icon), a higher proportion of eligible products in Singapore displayed the HCS (*n* = 86, 16%; *n* = 172, 34%, respectively). However, a larger proportion of Australian products displayed the HSR kJ icon (*n* = 188, 35%). The following categories had products displaying the HCS: ‘carbonated beverages’, ‘sports drinks’, ‘flavoured waters and cordials’, and ‘tea and coffee RTDs’. For these categories, a greater proportion of calorically sweetened products displayed the HCS compared with their non-calorically sweetened alternatives. 

Regardless of the country, none of the ‘drinking vinegar’ or ‘alcohol alternatives’ included in the analysis displayed either an HSR or HCS. In Australia, ‘sports drinks’ also did not display the HSR symbol, while in Singapore none of the ‘energy drinks’ were eligible to display the HCS.

## 4. Discussion

The aim of this paper was to provide a comparison of the market availability of sweetened or flavoured beverages between two countries: Australia and Singapore. Analysing differences and similarities between food supplies may be useful to facilitate consistent and effective food standards and regulations for a healthy global food supply. A baseline description of food supply composition prior to the implementation of policies can provide a benchmark to assess their effectiveness. This is particularly important in the context of food categories that are highly consumed and have significant detrimental impacts on human health, such as sugar-sweetened beverages [6,7,8,11]. 

### 4.1. Nutritional Differences between Beverage Categories and Countries

In this description of available sweetened and flavoured beverages, overall product counts were similar between countries. However, Singapore had a higher proportion of calorically sweetened beverages (88% vs. 70% of total products). These beverages had a higher median energy content than Australian products (134 ± 80 vs. 120 ± 98 kJ per 100 mL, *p* = 0.009). This may be explained by the high proportion of ‘Tea and coffee RTD’ and ‘Flavoured waters and cordial’ products in the Singaporean food supply, where Singaporean products had significantly higher median energy and sugar density. In fact, the median sugar content of calorically sweetened Singaporean ‘Tea and Coffee RTD’ products was 3.7 g/100 mL, or 276% higher than the median of Australian products. This was the second largest difference between groups, trumped only by the alcohol alternatives data, which were limited, as only one product with nutritional information was present in the Singaporean dataset. 

Current World Health Organization (WHO) guidelines recommend the consumption of no more than 10% of total daily energy from free sugars to mitigate health risks associated with their consumption, with added benefits if free sugar intake is below 5% of total daily energy [8]. Our findings indicate that just one serving of calorically sweetened carbonated beverages and energy drinks would be enough to exceed the WHO maximum daily free sugar recommendations in both countries (see Table 4). When looking at actual volumes, this was smaller than many canned beverages available in supermarkets (between 233 and 298 mLs). Thus, consumers could exceed the daily free sugar recommendations by consuming just one of these calorically sweetened beverages.

### 4.2. Nutrition Information Labels 

While a suite of effective policy strategies is required to minimise the impact of sweetened and flavoured beverages on human health, communication of the nutritional composition of products is a key first step. One hundred percent (100%) of the Australian products in the dataset provided on-pack nutrient information, as required by the Food Standards Code for Australia and New Zealand [44]. However, 7% of Singaporean products in the dataset did not provide on-pack energy information, and 10% did not display a total sugar value. On-pack nutrient information is not required in Singapore unless a health claim or nutrient content claim is made [45]. Because over 90% of the Singaporean food supply is imported [46], mandating consistent food labelling is a considerable challenge. In this analysis, it was noted that the language (English vs. Mandarin), format, and order of nutrients on the NIP were inconsistent, all of which are barriers to consumer use and interpretation. In both countries, many dietary recommendations, such as recommended limits for daily salt, sugar, or fat consumption, are easier to comply with if consumers are able to find and interpret nutrient information on food labels [47,48].

### 4.3. Use of Front-Of-Pack Labels 

In Australia, 52% of sweetened or flavoured beverages displayed either the HSR symbol or kJ icon, while 34% of sweetened or flavoured beverages in Singapore displayed the HCS. Both systems are voluntary. In the case of HSR, which allocates between 0.5 to 5 points (‘stars’), according to the quantity of ‘positive’ versus ‘risk’ nutrients per 100g of product [37], uptakes of FOPLs on low-scoring products has previously been limited [49]. This differs from the HCS which is a ‘binary’ system; i.e. the symbol is either displayed or not, depending on whether the product meets specific nutrient criteria [38].

Many products displayed HSR ratings that were low; the median HSR score for calorically sweetened products in the Australian dataset was 1.5 stars, which was paradoxical for a voluntary system. This may be related to manufacturers deciding to implement HSR ratings across their entire product ranges as part of their corporate social responsibility plans. Our findings showed that more Australian sweetened or flavoured beverages displayed the kJ icon, rather than the HSR symbol, which might reflect that energy content is perceived as less of a negative than a low HSR value. The energy icon is no longer in use due to poor consumer understanding [49]. 

It is conceivable that consumers would preferably purchase foods that carry a nutrient signposting FOPL, and thus they hold the potential to shift dietary consumption patterns. Australia does not have plans to require the use of the HSR on any categories of foods. However, the Singaporean Government has announced that it will be supporting the mandatory implementation of Nutri-Grade, an FOPL, for all categories of beverages [50]. This has been discussed previously in a related paper by Tan et al. [33].

### 4.4. Strengths and Limitations

A key strength of this study was the use of the FoodTrack^TM^ database and methodology in both countries. The real-time, technology-based, in-store data collection by trained nutrition researchers with auditing of all collected data is unique to the FoodTrack^TM^ methodology and enables high-quality data collection. FoodTrack^TM^ has been used to measure HSR uptake and compliance to both the HSR calculation and the display of the HSR symbol [51] as well as to inform improvements to the system [52]. However, some limitations remain. For example, Australian data were only collected from metropolitan Victoria, and thus the dataset is unlikely to include all products that are available across Australia. Despite these limitations, the data are still likely to represent a large proportion of the beverages available in Australian supermarkets, as 80% of Australian supermarket revenue is shared between the four companies included in our dataset [40]. Similarly, in Singapore, many of the local delis and small convenience stores were not surveyed, but market representation was estimated to be over 80% [33].

Choices were made about the beverage types to be categorised as sweetened and flavoured beverages, and plain water, alcoholic beverages, fruit juices, and beverages where milk and milk alternatives were a major component were excluded. It is recognised that beverages make significant contributions to multiple nutrients for Australians [53]. The focus of this paper was discretionary, or non-recommended beverages, and excluded alcoholic beverages, which are considered a special case. It is possible that the exclusion of milk-based beverages may have differentially removed more beverages from the Tea and Coffee RTD category in Australia than in Singapore because milk beverages (including coffee-flavoured beverages) are more widely available in Australia [54]. 

Unfortunately, due to the COVID-19 pandemic, data collection in Australia was discontinued in 2020. Thus, pre-pandemic data for the Australian food supply were compared with data for the Singaporean food supply that were collected during the early stages of the pandemic. The impacts of the pandemic on data collection are unknown. 

As previously mentioned, a small percentage (7%) of the products from Singapore did not display nutrient information. The lack of NIP data made food supply comparisons more challenging because the results were not necessarily representative of all available products. Despite this limitation, the collected data were still the most comprehensive nutritional composition data available for the categories collected within the Singaporean food supply. 

It is acknowledged that the supermarket availability of food does not accurately represent consumption. However, being able to easily access foods is a prerequisite for regular consumption. Therefore, various aspects of the supermarket food supply are relevant to health maintenance or improvement through better dietary intake.

Finally, this study only analysed energy and sugar. However, recent evidence suggests that factors beyond nutrients contribute to the healthfulness of food products [55]. While data on these food components are limited, future analyses could consider markers of ultra-processing found on ingredient lists.

## 5. Conclusions

As international food systems expand and overlap, analysing differences and similarities between food supplies is essential to facilitate consistent and effective food standards and regulations for a healthy global food supply. The aim of this paper was to provide a comparison of the market availability of sweetened or flavoured beverages between two countries: Australia and Singapore. Published data can also provide baseline evidence of food supply composition prior to the implementation of policies. This is particularly important in the context of food categories that are highly consumed and have significant detrimental impacts on human health, such as sugar-sweetened beverages. 

In this study, Singapore had proportionately more calorically sweetened beverages than Australia, and the median energy of calorically sweetened beverages was higher in Singapore than in Australia. About a third of sweetened or flavoured beverages collected in Singapore displayed the Healthy Choice Symbol, while in Australia 16% of included beverages showed the Health Star Rating symbol and 35% showed the now defunct kJ icon. These findings indicate that the consumption of just one serving of calorically sweetened carbonated beverages and energy drinks would exceed the WHO maximum daily free sugar recommendations.

Abbreviations: CSIRO: Commonwealth Scientific and Industrial Research Organisation; FOPL: Front-of-pack label; HCS: Healthy Choice Symbol; HSR: Health Star Rating; IQR: Inter-quartile Range; NIP: Nutrition Information Panel; NNS: Non-nutritive sweeteners; RTD: Ready-to-drink; WHO: World Health Organization.

## Figures and Tables

**Table 1:** Definitions and product examples for categories and subcategories of included beverages.

Category Name	Definition and Examples
Category	
Energy drinks	Carbonated or non-carbonated beverages marketed as an ‘energy enhancer’.
Sports drinks	Non-carbonated drinks marketed as a ‘sports enhancer’, often contain added vitamins and minerals.
Teas and Coffees	Teas and coffees where milk is not the primary ingredient. May or may not be carbonated. Include iced tea, ready-to-drink black coffee, and kombucha.
Carbonated beverages	All sweetened or flavoured carbonated beverages (except for tea products). Include creaming sodas, lemonades, lemon, lime, bitters, sarsaparilla, other soft drinks of any flavour, frozen soft drinks of any flavour, tonic water, club soda, and flavoured sparkling water.
Drinking Vinegar	Vinegar-based beverages, including switchel.
Flavoured waters and cordials	Flavoured non-carbonated beverages not classified elsewhere. Include cordial concentrates, ready-to-drink cordials, syrups, vitamin waters, coconut water, fruit-flavoured drinks, and still water with flavouring.
Alcohol alternatives	Non-alcoholic versions of traditionally alcoholic beverages, such as alcohol-free beer, wine, and cider.
Subcategory	
Calorically sweetened	Products with sugar listed in the ingredients in any form (other than fruit juice/fruit juice concentrate) that contain >1 g of sugar per 100 g. Include products that contain both caloric sweeteners and non-nutritive sweetening agents.
Non-calorically sweetened	Products with <1 g of sugar per 100 g. These products may have been sweetened using a non-nutritive sweetening agent. Excludes unflavoured products such as plain water.

**Table 2:** Numbers and proportions of beverage products collected within each category and subcategory in Australia (2019) and Singapore (2020).

Category and Subcategory	Australia (*n* = 531)		Singapore (*n* = 517)		Between-Country Difference
	*n*	%	*n*	%	*p*-Value
**Carbonated beverages**	215	40	131	25	<0.001 *
*Calorically sweetened*	135	63	106	81	<0.001 *
*Not calorically sweetened*	80	37	25	19	
**Energy drinks**	30	6	18	3	0.061
*Calorically sweetened*	22	73	15	83	^+^
*Not calorically sweetened*	8	27	3	17	
**Sports drinks**	46	9	25	5	0.006 *
*Calorically sweetened*	37	80	22	88	^+^
*Not calorically sweetened*	9	20	3	12	
**Flavoured waters and cordials**	132	25	158	30	0.358
*Calorically sweetened*	104	79	156	99	<0.001 *
*Not calorically sweetened*	28	21	2	1	^+^
**Tea and coffee RTDs**	93	18	182	35	<0.001 *
*Calorically sweetened*	66	71	155	85	0.008 *
*Not calorically sweetened*	27	29	27	15	
**Drinking vinegar**	4	1	8	2	^+^
*Calorically sweetened*	0	0	7	88	^+^
*Not calorically sweetened*	4	100	1	13	^+^
**Alcohol alternatives**	11	2	1	<1	^+^
*Calorically sweetened*	10	91	1	100	^+^
*Not calorically sweetened*	1	9	0	0	^+^
**Total**	531	100	523	100	
*Calorically sweetened*	374	70	462	88	<0.001 *
*Not calorically sweetened*	157	30	61	12	

Note: data include only unique products with NIPs presented ‘as consumed’; * statistically significant, as calculated by chi-squared tests; ^+^ unable to complete test due to an expected cell count of less than 5.

**Table 3:** Nutritional compositions (/100 mL) of calorically sweetened beverage products collected within each subcategory in Australia (2019) and Singapore (2020), showing the number of beverages (n) and the median and IQR values for energy and sugar.

	Energy (kJ per 100 mL)							Sugar (g per 100 mL)						
	Australia			Singapore				Australia			Singapore			
	*n*	Median	IQR	*n*	Median	IQR	*p*-Value	*n*	Median	IQR	*n*	Median	IQR	*p*-Value
Carbonated beverages	135	170 ^a,b,c^	98.0	102	152 ^j^	90.4	0.130	135	9.8 ^a,b,c^	6.0	99	8.6 ^j,k^	5.4	0.114
Energy drinks	22	195 ^a,d,e,f,g^	22.0	15	193 ^k,l,m^	140.1	0.963	22	11.0 ^a,d,e,f,g^	2.1	13	8.6 ^l,m^	7.1	0.442
Sports drinks	37	104 ^d,h^	22.0	22	101 ^j,k,n^	13.0	0.009	37	6.0 ^d,h^	0.2	22	5.7 ^j,l^	0.6	0.088
Flavoured waters and cordials	104	111 ^b,e,i^	57.0	136	143 ^n,o^	76.4	<0.001	104	5.7 ^b,e,i^	3.3	131	7.5 ^n^	4.3	<0.001
Tea and Coffee RTD	66	63 ^c,f,h,i^	46.0	147	105 ^l,o^	78.0	<0.001	66	2.1 ^c,f,h,i^	3.4	137	5.8 ^k,m,n^	3.3	<0.001
Drinking vinegar	0	-	-	7	115 ^m^	118.4	N/A	0	-	-	7	5.9	6.7	N/A
Alcohol alternatives	10	107 ^g^	97.0	1	184	0.0	0.343	10	3.9 ^g^	6.6	1	10.8	0.0	0.205
Total	374	120	98.0	430	134	79.6	0.009	374	6.4	6.1	410	6.8	4.2	0.129

Note: a shared superscript indicates a statistically significant (*p* < 0.05) between-product-group difference (Kruskal–Wallis tests). N/A indicates no comparator, and therefore we could not compute the statistic.

**Table 4:** Sugar per serving of calorically sweetened beverages in Australia and Singapore and their contributions to meeting/exceeding daily energy intakes from free sugars according to the World Health Organization guidelines.

		Australia						Singapore					
		% Contribution to WHO Guidelines per Serving		mLs of Drink Consumed to Exceed WHO Guidelines		Servings Taken to Exceed WHO Guidelines		% Contribution to WHO Guidelines per Serving		mLs of Drink Consumed to Exceed WHO Guidelines		Servings Taken to Exceed WHO Guidelines	
		Median	IQR	Median	IQR	Median	IQR	Median	IQR	Median	IQR	Median	IQR
Contribution to WHO guideline of 10% dietary energy from free sugars	Carbonated beverages	50.3	32.2	522	575	2.0	2.1	50.3	28.0	595	548	2.0	1.7
	Energy drinks	64.0	49.8	465	86	1.6	1.0	48.8	38.1	595	376	2.1	1.9
	Sports drinks	29.8	39.7	853	29	3.4	2.1	26.4	5.1	898	95	3.8	0.7
	Flavoured waters and cordials	28.9	12.7	907	481	3.5	1.5	35.6	20.3	683	446	2.8	1.6
	Tea and Coffee	12.3	16.7	2327	2633	7.8	7.4	29.3	17.8	883	481	3.4	1.8
	Total	34.2	30.5	764	712	2.9	2.6	33.7	23.4	753	482	3.0	1.9
Contribution to WHO guideline of 5% dietary energy from free sugars	Carbonated beverages	100.6	64.5	261	288	1.0	1.1	100.6	55.9	298	274	1.0	0.9
	Energy drinks	127.9	99.6	233	43	0.8	0.5	97.7	76.3	298	188	1.0	0.9
	Sports drinks	59.6	79.3	427	15	1.7	1.0	52.7	10.2	449	47	1.9	0.3
	Flavoured waters and cordials	57.8	25.5	453	241	1.7	0.7	71.3	40.6	341	223	1.4	0.8
	Tea and Coffee	24.5	33.4	1164	1317	3.9	3.7	58.6	35.5	441	241	1.7	0.9
	Total	68.4	61.0	382	356	1.5	1.3	67.4	46.9	376	241	1.5	1.0

Notes: only includes calorically sweetened beverages where category product counts were >10; calculation of percent contribution to WHO free-sugar guidelines was based on a daily intake of 8700 kJ. Sugar per serving and serving sizes are displayed in Supplementary Table S3.

**Table 5:** Presence of symbols (Health Star Rating and Healthier Choice Symbol) displayed on the fronts of packs of beverages within each category and subcategory in Australia (2019) and Singapore (2020).

	Australia						Singapore	
	Displaying HSR Score		Displaying HSR kJ Icon		HSR Score		HCS Present	
	*n*	%	*n*	%	Median	IQR	*n*	%
**Carbonated beverages**	**38**	**18**	**94**	**44**	**2.0**	**1.0**	**34**	**26**
*Calorically sweetened*	23	61	57	61	1.0	0.5	27	79
*Not calorically sweetened*	15	39	37	39	2.0	0.0	7	21
**Energy drinks**	**1**	**3**	**15**	**50**	**1.0**	**0.0**	**N/A**	**N/A**
*Calorically sweetened*	1	100	10	67	1.0	0.0	N/A	N/A
*Not calorically sweetened*	0	0	5	33			N/A	N/A
**Sports drinks**	**0**	**0**	**39**	**85**			**21**	**84**
*Calorically sweetened*	0	0	30	77			20	95
*Not calorically sweetened*	0	0	9	23			1	5
**Flavoured waters and cordials**	**36**	**27**	**25**	**19**	**2.0**	**1.0**	**49**	**31**
*Calorically sweetened*	22	61	18	72	2.0	3.5	49	100
*Not calorically sweetened*	14	39	7	28	3.0	1.0	0	0
**Tea and Coffee RTD**	**11**	**12**	**15**	**16**	**2.0**	**0.5**	**68**	**37**
*Calorically sweetened*	11	100	13	87	2.0	0.5	52	76
*Not calorically sweetened*	0	0	2	13			16	24
**Drinking vinegar**	**0**	**0**	**0**	**0**			**0**	**0**
**Alcohol alternatives**	**0**	**0**	**0**	**0**			**0**	**0**
**Total**	**86**	**16**	**188**	**35**	**2.0**	**0.5**	**172**	**34**
*Calorically sweetened*	57	66	128	68	1.5	1.0	148	86
*Not calorically sweetened*	29	34	60	32	2.0	1.0	24	14

Percentages relate to the percent of total products displaying HSRs within each category or subcategory. HSR = Health Star Rating; HCS = Healthier Choice Symbol; RTD = ready-to-drink; products that were ineligible for HCS are listed as N/A.

## Data Availability

The FoodTrack™ data presented in this study are not publicly available for commercial reasons.

## Supplementary Materials

The following supporting information can be downloaded at: https://www.mdpi.com/article/10.3390/nu15010247/s1, Supplementary Figure S1: Overview of the number of Australian sweetened and flavoured beverages included at each stage of the project (data collection, cleaning and analysis). Supplementary Figure S2: Overview of the number of Singaporean sweetened and flavoured beverages included at each stage of the project (data collection, cleaning and analysis). Supplementary Table S1: Products excluded due to data only being available for dry/ undiluted versions of products (i.e. insufficient NIP data) or because they were available in multiple pack sizes and therefore duplicated. Supplementary Table S2: nutrient information broken down into categories and sub-categories. Supplementary Table S3: Sugar per serve and serve sizes for calorically sweetened beverages included in Australia and Singapore.
