# Supplementary material for: Oh So Sweet: A Comparative Investigation of Retail Market Composition of Sweetened and Flavoured Beverages in Singapore and Australia
Source: Nutrients. 2023 Jan 3;15(1):247. doi: 10.3390/nu15010247 (PMC9824729; doi:10.3390/nu15010247)
Supplement: Supplementary file 1 [file nutrients-15-00247-s001.zip › nutrients-2095439-supplementary.pdf]

Supplementary Material

Supplementary Figure S1: Overview of the number of Australian sweetened and flavoured beverages included at each stage of the project (data collection, cleaning and analysis)

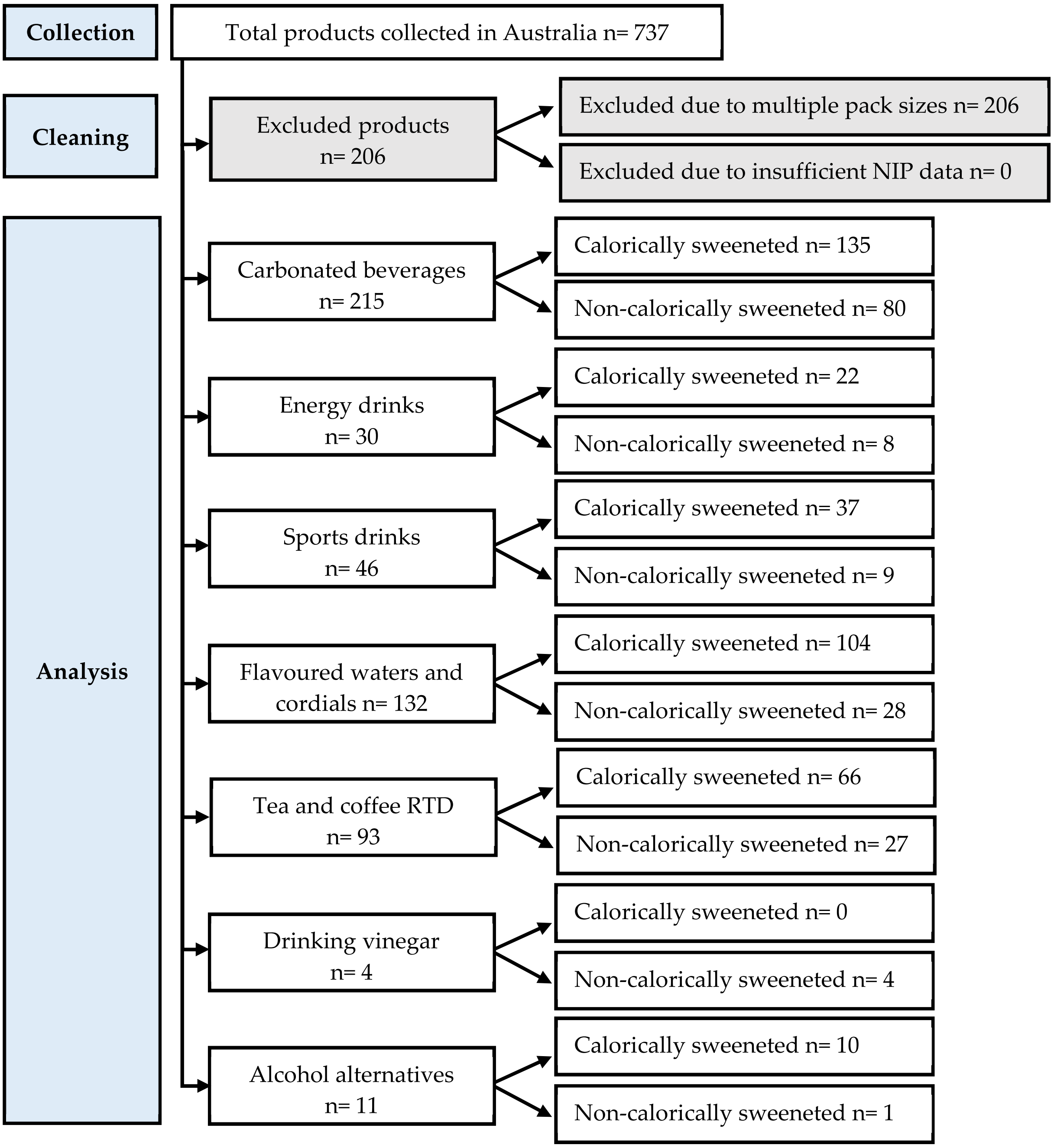

Supplementary Figure S2: Overview of the number of Singaporean sweetened and flavoured beverages included at each stage of the project (data collection, cleaning and analysis)

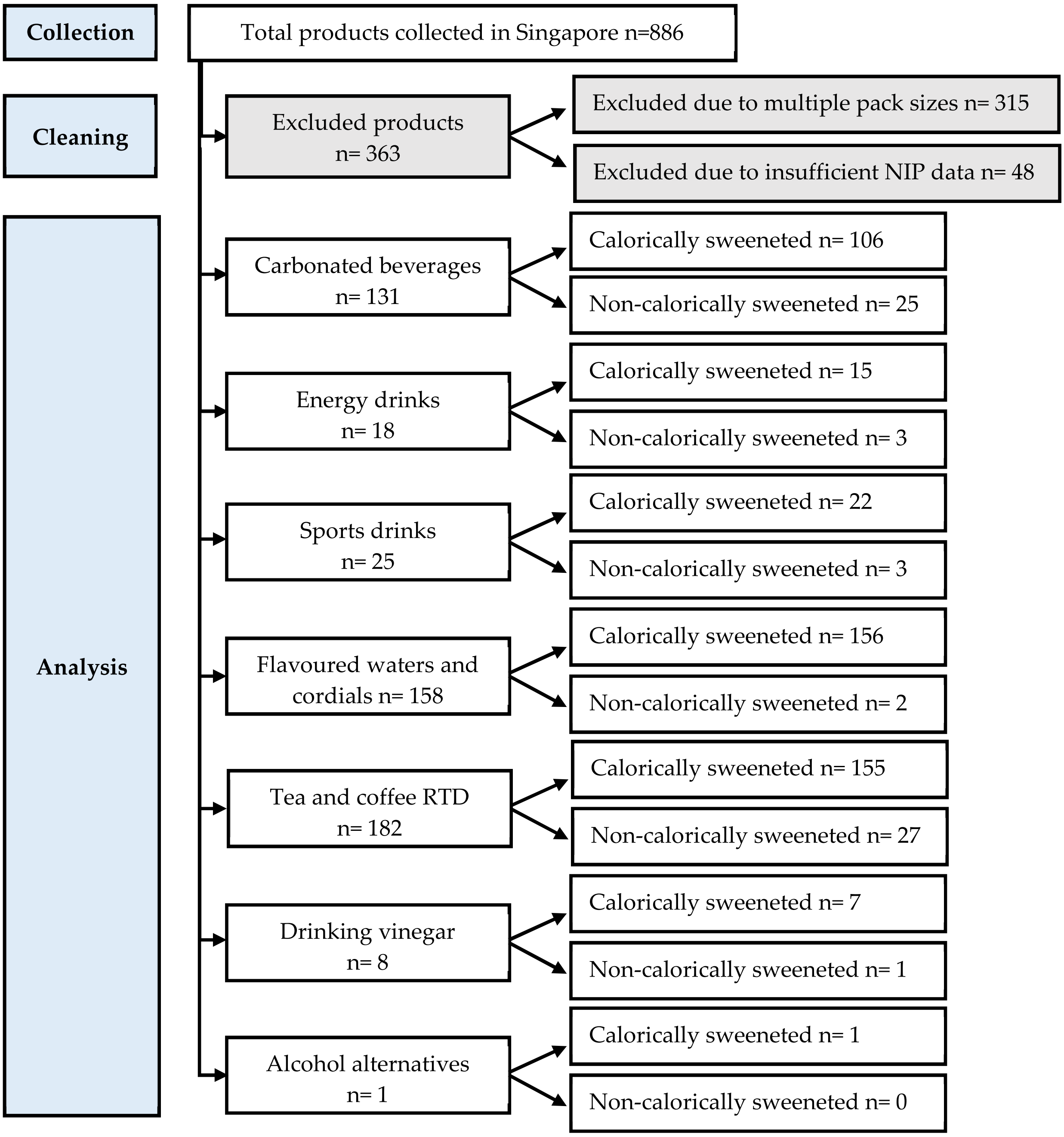

Supplementary Table S1: Products excluded due to data only being available for dry/ undiluted versions of products (i.e. insufficient NIP data) or because they were available in multiple pack sizes and therefore duplicated.

	Australia		Singapore		Total products excluded, n
	Dry/ undiluted, n	Size duplicate, n	Dry/ Undiluted, n	Size duplicate, n	
**Carbonated beverages**	-	121	-	90	211
Calorically sweetened	-	71	-	70	141
Not calorically sweetened	-	50	-	20	70
**Energy drinks**	-	23	-	9	32
Calorically sweetened	-	17	-	7	24
Not calorically sweetened	-	6	-	2	8
**Sports drinks**	-	8	3	31	42
Calorically sweetened	-	8	3	25	36
Not calorically sweetened	-	-	-	6	6
**Flavoured waters and cordials**	-	12	6	60	78
Calorically sweetened	-	12	6	60	78
Not calorically sweetened	-	-	-	-	-
**Tea and Coffee RTD**	-	38	9	122	169
Calorically sweetened	-	19	9	93	121

Not calorically sweetened	-	19	-	29	48
**Drinking vinegar**	-	4	30	3	37
Calorically sweetened	-	-	28	3	31
Not calorically sweetened	-	4	2	-	6
**Alcohol alternatives**	-	-	-	-	-

Supplementary Table S2: nutrient information broken down into categories and sub-categories.

	Energy (kJ per 100mL)						Sugar (g per 100mL)					
	Australia			Singapore			Australia			Singapore		
	Count	Median	IQR	Count	Median	IQR	Count	Median	IQR	Count	Median	IQR
**Carbonated beverages**												
Calorically sweetened	135	170	98.0	102	152	90.4	135.0	9.8	6.0	99.0	8.6	5.4
Not calorically sweetened	80	3	5.1	22	0	3.0	80.0	0.0	0.0	24.0	0.0	0.0
**Energy drinks**												
Calorically sweetened	22	195	22.0	15	193	140.1	22.0	11.0	2.1	13.0	8.6	7.1
Not calorically sweetened	8	13	1.6	3	12	107.7	8.0	0.0	0.0	3.0	0.0	0.0
**Sports drinks**												
Calorically sweetened	37	104	22.0	22	101	13.0	37.0	6.0	0.2	22.0	5.7	0.6
Not calorically sweetened	9	5	1.1	3	29	29.5	9.0	0.0	0.0	3.0	0.0	0.0

**Flavoured waters and cordials**												
Calorically sweetened	104	111	57.0	136	143	76.4	104.0	5.7	3.3	131.0	7.5	4.3
Not calorically sweetened	28	10	6.5	2	10	5.0	28.0	0.3	0.5	2.0	0.2	0.1
**Tea and Coffee RTD**												
Calorically sweetened	66	63	46.0	147	105	78.0	66.0	2.1	3.4	137.0	5.8	3.3
Not calorically sweetened	27	29	34.0	25	0	4.0	27.0	0.0	0.0	27.0	0.0	0.0
**Drinking vinegar**												
Calorically sweetened			0.0	7	115	118.4			0.0	7.0	5.9	6.7
Not calorically sweetened	4	45	22.5	1	0	0.0	4.0	0.0	0.0			0.0
**Alcohol alternatives**												
Calorically sweetened	10	107	97.0	1	184	0.0	10.0	3.9	6.6	1.0	10.8	0.0
Not calorically sweetened	1	91	0.0			0.0	1.0	1.0	0.0			0.0

**Total**												
Calorically sweetened	374	120	98.0	430	134	79.6	374.0	6.4	6.1	410.0	6.8	4.2
Not calorically sweetened	157	7	10.4	56	0	6.0	157.0	0.0	0.0	59.0	0.0	0.0

Supplementary Table S3: Sugar per serve and serve sizes for calorically sweetened beverages included in Australia and Singapore

	Australia				Singapore			
	Sugar per serve (g)		Serve Size (mL)		Sugar per serve (g)		Serve Size (mL)	
	Median	IQR	Median	IQR	Median	IQR	Median	IQR
**Carbonated beverages**	25.75	16.5	250	80	25.74	14.32	250	75
**Energy drinks**	32.75	25.5	290	250	25	19.52	250	10
**Sports drinks**	15.25	20.3	325	350	13.5	2.6	250	50
**Flavoured waters and cordials**	14.8	6.52	250	0	18.24	10.4	250	60
**Tea and Coffee**	6.27	8.54	250	80	15	9.1	250	50
**Total**	17.5	15.62	250	80	17.25	12	250	60
